# Association between variants of MTHFR genes and psychiatric disorders: A meta-analysis

**DOI:** 10.3389/fpsyt.2022.976428

**Published:** 2022-08-18

**Authors:** Yu-Xin Zhang, Lu-Ping Yang, Cong Gai, Cui-Cui Cheng, Zhen-yu Guo, Hong-Mei Sun, Die Hu

**Affiliations:** ^1^Department of Anatomy, School of Chinese Medicine, Beijing University of Chinese Medicine, Beijing, China; ^2^Department of Endocrinology, Guang’anmen Hospital, China Academy of Chinese Medical Sciences, Beijing, China; ^3^School of Chinese Materia Medica, Beijing University of Chinese Medicine, Beijing, China

**Keywords:** MTHFR C677T, MTHFR A1298C, disorders, meta-analysis, gene variants

## Abstract

**Background:**

Psychiatric disorders have seriously affected human life, one of the risk genes related to psychosis is the methylenetetrahydrofolatereductase (MTHFR) gene. This gene has a potential role in psychiatric disorders. Therefore, a meta-analysis is conducted to investigate the correlations between two prevalent MTHFR single nucleotide polymorphisms (SNPs), MTHFR C677T, A1298C, severe psychological disorders (schizophrenia, major depression, bipolar disorder).

**Methods:**

A total of 81 published studies were screened and selected by a search of electronic databases up to April 2022. Odds ratios (ORs) with 95% confidence intervals (CIs) were used to assess the association between MTHFR polymorphism and psychiatric disorders susceptibility by using random effect models.

**Results:**

We found that MTHFR C677T polymorphism is significantly related to schizophrenia and major depression in the overall population. MTHFR C677T has been linked to an increased risk of bipolar disorder in the recessive model (TT vs. CT + CC). Ethnic subgroup analysis shows that schizophrenia and major depression significantly correlate with MTHFR C677T and A1298C in Asian populations but not Caucasians. Besides, schizophrenia is correlated substantially with MTHFR C677T in the African population. However, the MTHFR A1298C polymorphism is only marginally linked to major depression.

**Conclusion:**

Findings of the current study revealed that MTHFR may contribute to the common pathogenesis of psychiatric diseases and that its variants may be essential in controlling the expression of psychosis-related genes. This study could help the researchers and health specialists in the early diagnosis and treatment of psychiatric disorders.

## Introduction

Mental disorders have seriously affected human life, causing considerable familial and social burden (1). They are among the leading causes of disability globally and have been related to an increase in premature mortality (2). Major psychiatric disorders include schizophrenia (SZ), major depression (MD), bipolar disorder (BPD), and others (3). These mental disorders are more likely to occur in families, suggesting that they are related to genetic factors (4, 5). Many susceptible genes have been found through unbiased genome-wide association studies (GWAS), a kind of analysis comparing allele frequencies of all available polymorphic markers with specific symptoms or disease states (6, 7). GWAS and many other follow-up replication studies have suggested that methylenetetrahydrofolatereductase (MTHFR) polymorphisms are associated with psychiatric disorders.

The MTHFR is a crucial enzyme in the one-carbon metabolism (OCM) process, which involves folate and homocysteine (Hcy) metabolisms. It transforms 5,10-methylenetetrahydrofolate (5,10-methylene THF) to 5-methyltetrahydrofolate (5-methyl THF), and it is involved in folate and homocysteine conversion, which is linked to DNA methylation (8–10). A number of mutations in the MTHFR gene have been found, and the most common mutations are C677T (rs1801133) and A1298C (rs1801131), which are correlated with enzyme deficiency (11–14). In addition, MTHFR polymorphism may significantly decrease MTHFR activity, affect the concentration of Hcy in plasma, and lead to a wide range of mental, neurological, and vascular dysfunction (15).

The human Methylenetetrahydrofolatereductase (MTHFR) gene is located in chromosomal region 1p36.3 (16). The MTHFR gene has 14 common or rare single nucleotide polymorphisms linked with enzyme defects, the most prevalent of which are C677T and A1298C. The C677T gene location is one of the most researched and clinically significant variants in exon 4. The variation in C677T is due to the replacement of cytosine by thymine, which leads to the conversion of valine to alanine at codon 222 (11). The polymorphism of A1298C is due to the adenine substitution by cytosine, leading to the conversion of glutamic acid to alanine at residue 429 (10). The replacement of 677 and 1,298 nucleotides C-T and A-C in the MTHFR gene reduces enzyme activity, and this decrease in MTHFR activity may affect the OCM cycle (17). Abnormal OCM might impair cortical and hippocampal neurogenesis during development and affect brain maturation and function (18–20).

The association between MTHFR polymorphism and mental illnesses has already been explored, but the influence of MTHFR on psychiatric disorders is still disputed, and limited studies have been found (21–23). These inconsistencies might be attributed to limited sample sizes, ethnic heterogeneity, and differences in population substructure. So, in current study these limitations have been overcome and summarized the conflicting data. A meta-analysis is performed to explore the connection of MTHFR C677T and A1298C polymorphisms with major mental disorders (including SZ, MD, and BPD). We also assessed whether ethnicity would affect the results. Therefore, it will provide more powerful evidence of whether MTHFR variants influence psychiatric diseases.

## Materials and methods

### Search strategy

We initially searched PubMed, Embase, Proquest, Web of Science, CNKI (Chinese National Knowledge Infrastructure), VIP (Chinese) database, and Wanfang (Chinese) database for the following terms: MTHFR (methylenetetrahydrofolatereductase), gene (gene or genetic or polymorphism or variants or variation), and psychiatric disorders (psychiatry disorders or mental illness or mental disorders or psychosis). We discovered that most research concentrated on MTHFR C677T and MTHFR A1298C. The researchers investigated the relationships between MTHFR gene variants and susceptibility to mental diseases such as schizophrenia, bipolar disorder, and depression. To guarantee that we missed no studies, we searched these databases again using these gene terms (MTHFR C677T and A1298C) and major mental disorders such as “schizophrenia,” “bipolar disorder,” “depression,” and so on. All of the research was completed and published by April 2022. After that, we selected relevant papers and examined their bibliographies to find additional references.

### Study selection

Selection of articles for analysis purposes was made based on the following criteria: (1) case-control studies; (2) giving comprehensive data of formally diagnosed patients with unrelated healthy control subjects for generating an odds ratio (OR) with a 95% confidence interval (CI); (3) Case status was classified as having a DSM-IV-diagnosed mental condition, with control patients having no history of psychiatric disorders or other neurological abnormalities; (4) the studies used samples that did not overlap with other studies; (5) the use of internationally recognized loci gene polymorphism detection techniques (such as polymerase chain reaction-restriction fragment length polymorphism, real-time quantitative polymerase chain reaction, or amplification block mutation system-polymerase chain reaction); and (6) the demographic characteristics of the control group, such as gender and age, were comparable to those of the case group. In addition, articles were excluded if they (1) not reported the target genotype frequencies, (2) were reviews, letters, or commentaries, or (3) were duplicate reports.

### Data extraction and management

Two reviewers independently extracted the following information from all eligible studies: author, year of publication, country, ethnicity (categorized as Asian, Caucasian, and African populations), and the number of distinct genotypes in cases and controls for C677T or A1298C genotype. In the case of a disagreement, a discussion was held, and if no agreement could be achieved, a third person was consulted for consensus.

### Statistical analysis

We investigated the potential of conducting a meta-analysis of all eligible studies. The odds ratio (OR) and associated 95% confidence intervals (CIs) were used to examine the strength of the connection between MTHFR polymorphism and mental disorders: the allele model (T vs. C, C vs. A), the dominant model (TT + CT vs. CC, CC + AC vs. AA), the homozygote model (TT vs. CC, CC vs. AA) and the recessive model (TT vs. CT + CC, CC vs. AC + AA). The Chi-square test was used to analyze the genotype distribution in the control groups for Hardy Weinberg equilibrium (HWE). The Cochran’s (*Q*) *X*^2^ test and *I*^2^ statistic were used to assess the heterogeneity between individual studies (24). Considering the heterogeneity of studies, this meta-analysis adopted a random effect model (25). Subgroup analyses were performed using ethnicity stratification, and sensitivity analyses were undertaken by excluding papers from the meta-analysis that were not in HWE. The funnel plots were displayed and evaluated using Egger’s linear regression test to control publication bias (26). Stata 14.0 was used to conduct all statistical analyses (StataCorp, College Station, TX, United States). A *P*-value of less than 0.05 was regarded as statistically significant. The article mainly showed the forest plots of T vs. C of MTHFR C677T and C vs. A of MTHFR A1298C; the other results were shown in the tables.

## Results

### Characteristics of eligible studies

**FIGURE 1:**
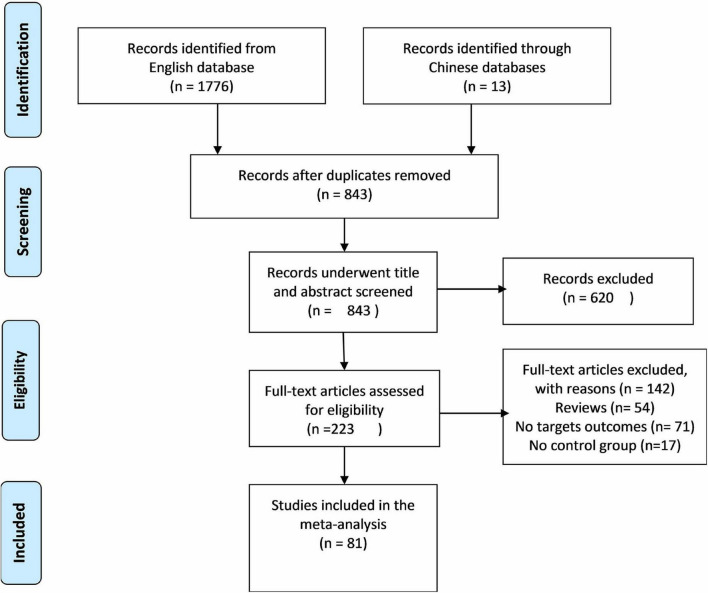
Flow diagram of the study selection process.

Out of screened articles, 843 unduplicated association studies were found. Figure 1 depicts a flow chart of the research process, the eliminated studies, and the reasons for their exclusion. Following an initial literature search and further screening, 81 (27–106) publications were retrieved. Our meta-analysis comprised 49,775 subjects (20,981 patients and 28,794 controls) with MTHFR C677T genotyping and 16,058 subjects (6,690 patients and 9,368 controls) with MTHFR A1298C genotyping. Detailed information (first author, year of publication, country, ethnicity, case/control, genotype, and P_*HWE*_) of included articles are summarized in Tables 1, 2.

**TABLE 1:** Overview of MTHFR C677T genotype distribution of psychosis patients and controls, with information about country, ethnicity, and disease.

References	Year	Country	Ethnicity	Case	Control	Case		Control		*P*_*HWE*_		
						CC	CT	TT	CC	CT	TT	
**Schizophrenia**												
Arinami et al. (27)	1997	Japanese	Asian	297	419	96	138	63	154	214	51	0.074
Kunugi et al. (28)	1998	Japanese	Asian	343	258	121	168	54	95	129	34	0.342
Virgos et al. (29)	1999	Spain	Caucasian	210	218	81	98	31	79	106	33	0.793
Joober et al. (30)	2000	Canada	Caucasian	105	90	30	52	23	41	36	13	0.278
Sazci et al. (31)	2003	Turkey	Caucasian	130	226	59	49	22	106	103	17	0.236
Tan et al. (32)	2004	Singapore	Asian	236	120	136	84	16	80	33	7	0.165
Yu et al. (33)	2004	China	Asian	230	251	91	96	43	85	126	40	0.554
Yu et al. (33)	2004	Scotland	Caucasian	426	628	199	186	41	306	260	62	0.535
Sazci et al. (34)	2005	Turkey	Asian	297	341	144	115	38	161	156	24	0.093
Vilella et al. (35)	2005	Spain	Caucasian	158	234	58	75	25	85	85	39	0.952
Kempisty et al. (36)	2006	Poland	Caucasian	200	300	113	68	19	210	79	11	0.303
Philibert et al. (37)	2006	United States	Caucasian	206	359	107	83	16	176	137	46	0.021*
Lee et al. (38)	2006	South Korea	Asian	235	235	74	128	33	99	115	21	0.009*
Yang et al. (39)	2007	China	Asian	100	100	33	51	16	52	40	8	0.937
Jonsson et al. (40)	2008	Denmark	Caucasian	419	1006	200	177	42	490	413	103	0.249
Jonsson et al. (40)	2008	Norway	Caucasian	163	177	75	70	18	80	75	22	0.501
Jonsson et al. (40)	2008	Sweden	Caucasian	258	293	137	104	17	156	113	24	0.581
Muntjewerff (41)	2008	Netherlands	Caucasian	252	405	110	111	31	205	165	35	0.61
Roffman et al. (42)	2008	United States	Caucasian	79	75	41	27	11	35	32	8	0.865
Feng et al. (43)	2009	China	Asian	123	123	17	67	39	40	65	18	0.308
Betcheva et al. (44)	2009	Bulgaria	Caucasian	185	182	76	85	24	84	76	22	0.457
García-Miss et al. (45)	2010	Mexico	Caucasian	105	108	29	45	31	22	54	31	0.864
Kang et al. (46)	2010	Korean	Asian	360	348	125	176	59	130	158	60	0.317
Ye et al. (47)	2010	China	Asian	104	56	12	58	34	14	32	10	0.266
Bouaziz et al. (48)	2010	Tunisia	African	25	25	18	4	3	19	5	1	0.397
Arzaghi et al. (49)	2011	Iran	Asian	66	94	35	27	4	54	38	2	0.11
Kim et al. (50)	2011	Korean	Asian	201	350	62	101	38	112	167	71	0.313
Muntjewerff et al. (51)	2011	Netherlands	Caucasian	739	886	334	319	86	405	389	92	0.921
Tsutsumi et al. (52)	2011	Japan	Asian	413	385	160	184	69	138	183	64	0.8
Zhang et al. (53)	2012	China	Asian	235	102	96	113	26	52	45	5	0.225
Lochman et al. (54)	2013	Czechia	Caucasian	186	209	72	90	24	105	86	18	0.948
Zhang et al. (55)	2013	China	Asian	1002	1036	166	450	384	213	505	318	0.63
Kontis et al. (56)	2013	Greece	Caucasian	90	55	40	37	13	21	22	12	0.187
El-Hadidy et al. (57)	2014	Egypt	African	103	149	52	36	15	114	30	5	0.103
Hei et al. (58)	2014	China	Asian	130	80	17	65	48	24	38	18	0.029
Nishi et al. (59)	2014	Japan	Asian	621	486	220	309	92	174	239	73	0.532
Nishi et al. (59)	2014	Japan	Asian	1,149	2,742	417	530	202	1,072	1,260	410	0.207
Foroughmand et al. (60)	2015	Iran	Asian	200	200	104	76	20	123	64	13	0.244
Misiak et al. (61)	2016	Poland	Caucasian	135	146	64	52	16	71	53	22	0.786
Takano et al. (62)	2016	Japan	Asian	45	30	17	18	10	12	14	4	0.62
Wang et al. (63)	2017	China	Asian	254	339	79	129	46	109	175	55	0.26
Oniki et al. (64)	2017	Japan	Asian	256	194	89	135	32	64	93	37	0.207
Debost et al. (65)	2017	Denmark	Caucasian	1699	1681	839	704	156	829	724	128	0.08
Zhilyaeva et al. (66)	2018	Russia	Caucasian	500	499	245	212	43	280	188	31	0.057
Ota et al. (67)	2019	Japan	Asian	538	1263	181	255	102	458	604	201	0.937
Wan et al. (68)	2019	China	Asian	97	92	24	47	26	24	43	25	0.532
Wan, L (69)	2019	China	Asian	242	234	45	122	75	71	113	50	0.687
**Major depression**												
Arinami et al. (27)	1997	Japanese	Asian	32	419	9	14	9	154	214	51	0.074
Kunugi et al. (28)	1998	Japanese	Asian	71	258	10	31	30	95	129	34	0.342
Tan et al. (32)	2004	Singapore	Asian	88	120	49	34	5	80	33	7	0.165
Kelly et al. (70)	2004	United Kingdom	Caucasian	100	89	30	56	14	40	37	12	0.467
Reif et al. (71)	2005	Germany	Caucasian	46	176	23	17	6	75	80	21	0.962
Yuan et al. (72)	2005	China	Asian	60	80	22	27	11	27	38	15	0.801
Chen-Sheng et al. (73)	2005	China	Asian	39	20	22	15	2	11	9	0	0.194
Yuan (74)	2007	China	Asian	60	80	22	27	11	27	38	15	0.801
Słopien et al. (75)	2008	Poland	Caucasian	83	89	26	38	19	46	36	7	0.991
Zhao (76)	2008	China	Asian	77	85	12	37	28	21	48	16	0.219
Yuan et al. (77)	2008	China	Asian	116	80	46	48	22	27	38	15	0.801
Hong et al. (78)	2009	China	Asian	178	85	75	84	19	32	44	9	0.28
Kim et al. (79)	2009	China	Asian	63	458	16	28	19	84	248	126	0.63
Pan et al. (80)	2009	United States	Caucasian	170	83	72	79	19	30	44	9	0.598
Cao et al. (81)	2010	China	Asian	50	59	9	23	18	24	27	8	0.926
Zeman et al. (82)	2010	Czechia	Caucasian	42	41	15	18	9	16	17	8	0.377
Feng et al. (83)	2010	China	Asian	152	152	32	66	54	51	81	20	0.167
Li et al. (84)	2010	China	Asian	402	600	132	192	78	156	343	101	<0.001*
Song (85)	2010	China	Asian	156	123	33	68	55	35	74	14	0.008*
Lizer et al. (86)	2011	United States	Caucasian	82	74	31	34	17	33	28	13	0.114
Zhao et al. (87)	2011	China	Asian	94	98	24	43	27	36	45	17	0.651
Chojnicka et al. (88)	2012	Poland	Caucasian	710	2547	342	300	68	1213	1081	253	0.593
Evinova et al. (89)	2012	Slovak	Caucasian	134	143	70	54	10	58	73	12	0.1
Qiao et al. (90)	2012	China	Asian	94	98	24	43	27	36	45	17	0.651
Shen et al. (91)	2014	China	Asian	368	219	88	259	21	113	91	15	0.563
Sayadi et al. (92)	2016	Tunisia	African	208	187	105	80	23	80	93	14	0.066
Mei et al. (93)	2016	China	Asian	37	65	9	26	2	32	27	6	0.59
Huang et al. (94)	2017	China	Asian	80	80	20	36	24	30	38	12	0.995
Li et al. (95)	2017	China	Asian	218	582	97	93	28	461	89	32	<0.001*
Mei et al. (96)	2018	China	Asian	106	175	25	75	6	90	73	12	0.59
Saraswathy et al. (97)	2019	India	African	91	206	78	12	1	183	22	1	0.68
**Bipolar disorder**												
Arinami et al. (27)	1997	Japanese	Asian	40	419	15	20	5	154	214	51	0.074
Kunugi et al. (28)	1998	Japanese	Asian	143	258	41	74	28	95	129	34	0.342
Tan et al. (32)	2004	Singapore	Asian	167	120	99	60	8	80	33	7	0.165
Reif et al. (71)	2005	Germany	Caucasian	92	176	48	34	10	75	80	21	0.962
Kempisty et al. (36)	2006	Poland	Caucasian	200	300	108	73	19	210	79	11	0.303
Zhao et al. (98)	2008	China	Asian	61	73	12	28	21	18	40	15	0.404
Ozbek et al. (99)	2008	Turkey	Caucasian	197	238	104	76	17	116	97	25	0.603
Jonsson et al. (40)	2008	Norway	Caucasian	117	177	58	49	10	80	75	22	0.501
Chen et al. (100)	2009	China	Asian	501	461	178	231	92	153	235	73	0.272
Ezzaher et al.(101)	2011	Tunisia	African	92	170	41	40	11	94	62	14	0.411
Arzaghi et al. (49)	2011	Iran	Asian	90	94	52	34	4	54	38	2	0.11
El-Hadidy et al. (57)	2013	Egypt	African	134	149	46	70	18	114	30	5	0.239
Permoda-Osip et al. (102)	2014	Poland	Caucasian	112	164	51	50	11	66	82	16	0.657
Wang et al. (103)	2015	China	Asian	531	447	287	206	38	215	199	33	0.16
Rahimi et al. (104)	2016	Iran	Caucasian	150	148	69	67	14	81	62	5	0.093

*P < 0.05.

**TABLE 2:** Overview of MTHFR A1298C genotype distribution of psychosis patients and controls, with information about country, ethnicity, and disease.

First author	Year	Country	Ethnicity	Case	Control	Case			Control			*P*_*HWE*_
						AA	AC	CC	AA	AC	CC	
**Schizophrenia**												
Sazci et al. (31)	2003	Turkey	Caucasian	130	226	57	59	14	114	93	19	0.996
Yu et al. (33)	2004	China	Asian	230	251	130	78	22	154	81	16	0.235
		Scotland	Caucasian	426	628	177	209	40	292	272	64	0.955
Sazc et al. (34)	2005	Turkey	Caucasian	297	341	130	129	38	159	155	27	0.201
Vilella et al. (35)	2005	Spain	Caucasian	158	234	76	68	14	124	97	13	0.286
Lee et al. (38)	2006	South Korea	Asian	235	236	157	7	71	145	14	77	<0.001*
Kempisty et al. (105)	2007	Poland	Caucasian	200	300	109	74	17	185	105	10	0.29
Jonsson et al. (40)	2008	Denmark	Caucasian	418	1004	184	186	48	462	419	123	0.052
	2008	Norway	Caucasian	163	177	89	60	14	82	79	16	0.625
	2008	Sweden	Caucasian	258	293	110	113	35	122	129	42	0.406
Betcheva et al. (44)	2009	Bulgaria	Caucasian	181	183	91	72	18	80	79	24	0.406
Kang et al. (46)	2010	Korean	Asian	360	348	248	105	7	239	100	9	0.703
Zhang et al. (106)	2010	China	Asian	379	380	230	127	22	260	108	12	0.848
Kim et al. (50)	2011	Korean	Asian	201	350	129	67	5	240	105	5	0.083
Zhang et al. (53)	2012	China	Asian	235	102	126	91	18	62	33	7	0.376
Foroughmand et al. (60)	2015	Iran	Asian	200	200	65	108	27	60	89	51	0.126
Misiak et al. (61)	2016	Poland	Caucasian	135	146	55	64	13	55	72	19	0.64
Takano et al. (62)	2016	Japan	Asian	45	30	34	8	3	21	9	0	0.2
Oniki et al. (64)	2017	Japan	Asian	256	194	173	75	8	124	65	5	0.597
Ota et al. (67)	2019	Japan	Asian	537	1262	358	163	16	820	395	47	0.947
Wan et al. (68)	2019	China	Asian	97	92	66	29	2	69	22	1	0.603
Wan et al. (69)	2019	China	Asian	242	234	174	63	5	171	58	5	0.975
**Major depression**												
Reif et al. (71)	2005	Germany	Caucasian	46	184	16	21	9	75	96	13	0.016*
Zeman et al. (82)	2010	Czechia	Caucasian	42	41	22	17	3	20	18	3	0.495
Feng et al. (83)	2010	China	Asian	152	152	122	28	2	115	35	2	0.716
Evinova et al. (89)	2012	Slovak	Caucasian	134	143	49	65	20	70	61	12	0.801
Li et al. (95)	2017	China	Asian	218	582	86	75	57	396	144	42	<0.001*
**Bipolar disorder**												
Reif et al. (71)	2005	Germany	Caucasian	92	184	30	47	15	75	96	13	0.016*
Kempisty et al. (105)	2007	Poland	Caucasian	200	300	99	78	23	185	105	10	0.29
Jonsson et al. (40)	2008	Norway	Caucasian	115	177	47	56	12	82	79	16	0.624
Ozbek et al. (99)	2008	Turkey	Caucasian	197	238	91	84	22	113	101	24	0.848
Permoda-Osip et al. (102)	2014	Poland	Caucasian	111	156	51	50	10	60	74	22	0.915

*P < 0.05.

### Methylenetetrahydrofolatereductase C677T/A1298C and psychiatric disorders

#### Association between the methylenetetrahydrofolatereductase C677T/A1298C polymorphisms and schizophrenia

**FIGURE 2:**
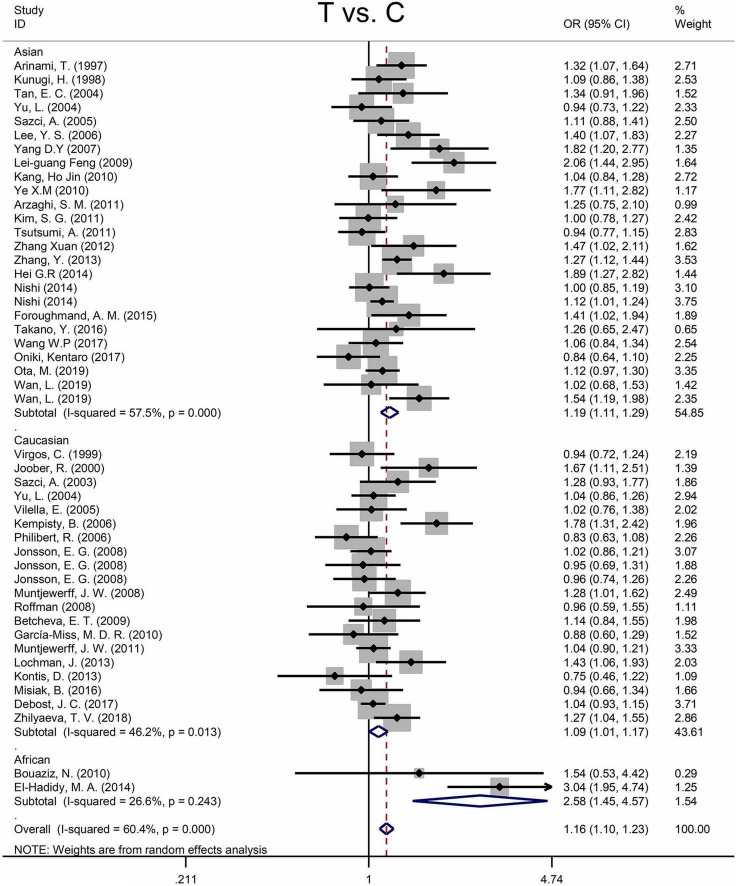
Forest plots for the associations between MTHFR C677T polymorphisms and SZ for the allele model with random effect model.

Findings of the association and the heterogeneity test is shown in Table 3. MTHFR C677T polymorphism was shown to be highly associated with an increased risk of developing SZ in all statistical models (for T vs. C, OR = 1.16, 95% CI = 1.10–1.23, *P* < 0.001; for TT + CT vs. CC: OR = 1.18, 95% CI = 1.10–1.27, *P* < 0.001; for TT vs. CT + CC: OR = 1.25, 95% CI = 1.13–1.37, *P* < 0.001; for TT vs. CC: OR = 1.35, 95% CI = 1.19–1.52, *P* < 0.001) (Figure 2 and Table 3).

**TABLE 3:** Odds ratios and heterogeneity results for the 4 genetic models of the MTHFR C677T and A1298C for SZ.

MTHFR		Comparison model	OR (95% CI)	*P*_*OR*_	Heterogeneity		
					*Q* within	*P*-value	*I*^2^ (%)
MTHFRC677T	All studies	T vs. C	1.16(1.10–1.23)	<0.001	116.30	<0.001	60.4
		TT + CT vs. CC	1.18(1.10–1.27)	<0.001	93.38	<0.001	50.7
		TT vs. CT + CC	1.25(1.13–1.37)	<0.001	80.44	0.001	42.8
		TT vs. CC	1.35(1.19–1.52)	<0.001	103.78	<0.001	55.7
	Asian	T vs. C	1.19(1.11–1.29)	<0.001	56.46	<0.001	57.5
		TT + CT vs. CC	1.22(1.10–1.35)	<0.001	48.36	0.002	50.4
		TT vs. CT + CC	1.31(1.16–1.48)	<0.001	41.63	0.014	42.3
		TT vs. CC	1.46(1.24–1.72)	<0.001	57.48	<0.001	58.2
	Caucasian	T vs. C	1.09(1.01–1.17)	0.036	35.29	0.013	46.2
		TT + CT vs. CC	1.11(1.01–1.21)	0.034	28.48	0.075	33.3
		TT vs. CT + CC	1.12(0.97–1.29)	0.132	27.76	0.088	31.6
		TT vs. CC	1.16(0.98–1.37)	0.082	32.08	0.031	40.8
	African	T vs. C	2.58(1.45–4.57)	0.001	1.36	0.243	26.6
		TT + CT vs. CC	2.37(1.00–5.64)	0.050	1.84	0.175	45.6
		TT vs. CT + CC	4.59(1.77–11.92)	0.002	0.10	0.756	0
		TT vs. CC	5.81(1.20–15.32)	<0.001	0.31	<0.001	0
MTHFR A1298C	All studies	C vs. A	1.04(0.96–1.13)	0.305	33.40	0.042	37.1
		CC + AC vs. AA	1.06(0.98–1.15)	0.165	23.60	0.313	11.0
		CC vs. AC + AA	1.05(0.88–1.25)	0.622	31.24	0.07	32.8
		CC vs. AA	1.08(0.89–1.29)	0.438	31.32	0.069	32.9
	Caucasian	C vs. A	1.05(0.95–1.17)	0.327	14.04	0.121	35.9
		CC + AC vs. AA	1.07(0.95–1.20)	0.289	11.04	0.273	18.5
		CC vs. AC + AA	1.09(0.87–1.37)	0.434	13.54	0.14	33.5
		CC vs. AA	1.12(0.88–1.44)	0.357	14.85	0.095	39.4
	Asian	C vs. A	1.03(0.92–1.16)	0.602	18.98	0.061	42.0
		CC + AC vs. AA	1.05(0.94–1.18)	0.418	12.42	0.333	11.5
		CC vs. AC + AA	1.00(0.74–1.34)	0.981	16.80	0.114	34.5
		CC vs. AA	1.02(0.77–1.37)	0.870	15.70	0.153	29.9

An ethnic subgroup analysis revealed a substantial association between MTHFR C677T polymorphism and SZ among Asian populations (for T vs. C: OR = 1.19, 95% CI = 1.11–1.29, *P* < 0.001; for TT + CT vs. CC: OR = 1.22, 95% CI = 1.10–1.35, *P* < 0.001; for TT vs. CT + CC: OR = 1.31, 95% CI = 1.16–1.48, *P* < 0.001; for TT vs. CC: OR = 1.46, 95% CI = 1.24–1.72, *P* < 0.001); in Caucasian populations, a significant association was found with the allele model (for T vs. C: OR = 1.09, 95% Cl = 1.01–1.17, *P* = 0.036) and the dominant model (for TT + CT vs. CC: OR = 1.11, 95% Cl = 1.01–1.21, *P* = 0.034); in African populations, there was a significant association with the allele model (for T vs. C: OR = 2.58, 95% Cl = 1.45–4.57, *P* = 0.001), the recessive model (TT vs. CT + CC: OR = 4.59, 95% CI = 1.77–11.92, *P* = 0.002) and the homozygote model (for TT vs. CC: OR = 5.81, 95% Cl = 1.20–15.32, *P* < 0.001). All these findings are summarized in Table 3. Subgroup analysis reveals that the association between MTHFR C677T polymorphism and SZ exists in Asian (all genetic models) and African populations (allele models, recessive models, and homozygous models) but not in Caucasian (only allele models and dominant models).

**FIGURE 3:**
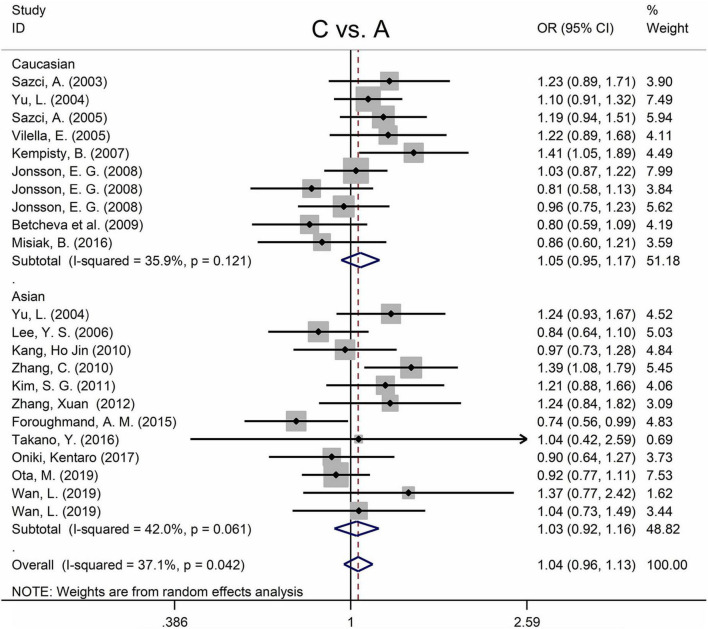
Forest plots for the associations between MTHFR A1298C polymorphisms and SZ for the allele model with random effect model.

The MTHFR A1298C polymorphism was not statistically correlated with SZ in all models (Figure 3 and Table 3). Moreover, subgroup analysis revealed no correlation between the MTHFR A1298C polymorphism and SZ in Asian or Caucasian populations (Figure 3 and Table 3). African populations were not included in the study because of the small number of studies.

There were two articles not in Hardy–Weinberg equilibrium (37, 38) (Tables 1, 2). Sensitivity analysis revealed that the overall association between MTHFR C677T polymorphism and SZ remained unchanged after omitting these two samples from the meta-analysis (for T vs. C: OR = 1.17, 95% CI = 1.10–1.24, *P* < 0.001, Supplementary Figure 6; for TT + CT vs. CC: OR = 1.18, 95% CI = 1.10–1.28, *P* < 0.001; for TT vs. CT + CC: OR = 1.25, 95% CI = 1.14–1.38, *P* < 0.001; for TT vs. CC: OR = 1.35, 95% CI = 1.20–1.53, *P* < 0.001). Sensitivity analysis for the MTHFR A1298C polymorphism revealed that excluding Lee et al. (38) had no impact on the conclusion of the meta-analysis (Supplementary Figure 7).

#### Association between the methylenetetrahydrofolatereductase C677T/A1298C polymorphisms and major depression

**FIGURE 4:**
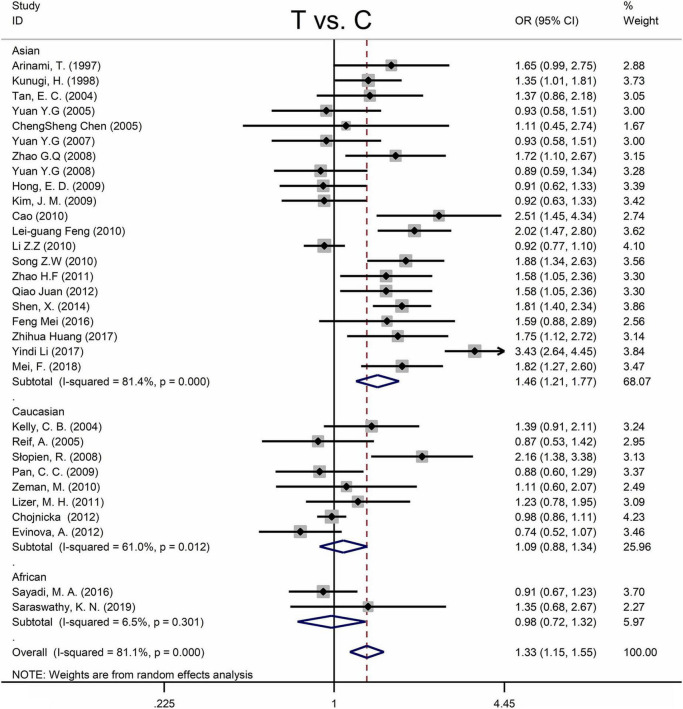
Forest plots for the associations between MTHFR C677T polymorphisms and MD for the allele model with random effect model.

Table 4 shows the main results as well as the heterogeneity test. MTHFR C677T polymorphism was shown to be highly associated with an increased risk of developing MD in all statistical models (for T vs. C: OR = 1.33, 95% CI = 1.15–1.55, *P* < 0.001; for TT + CT vs. CC: OR = 1.35, 95% CI = 1.08–1.70, *P* = 0.009; for TT vs. CT + CC: OR = 1.58, 95% CI = 1.28–1.95, *P* < 0.001; for TT vs. CC: OR = 1.66, 95% CI = 1.31–2.11, *P* < 0.001) (Figure 4 and Table 4).

**TABLE 4:** Odds ratios and heterogeneity results for the 4 genetic models of the MTHFR C677T and A1298C for MD.

MTHFR		Comparison model	OR (95% CI)	*P*_*OR*_	Heterogeneity		
					*Q* within	*P*-value	*I*^2^ (%)
MTHFRC677T	All studies	T vs. C	1.33(1.15–1.55)	<0.001	159.05	<0.001	81.1
		TT + CT vs. CC	1.35(1.08–1.70)	0.009	183.95	<0.001	83.7
		TT vs. CT + CC	1.58(1.28–1.95)	<0.001	75.2	<0.001	60.1
		TT vs. CC	1.66(1.31–2.11)	<0.001	80.47	<0.001	62.7
	Asian	T vs. C	1.46(1.21–1.77)	<0.001	107.45	<0.001	81.4
		TT + CT vs. CC	1.52(1.11–2.08)	0.009	135.03	<0.001	85.2
		TT vs. CT + CC	1.75(1.34–2.28)	<0.001	54.54	<0.001	63.3
		TT vs. CC	1.89(1.40–2.57)	<0.001	56.63	<0.001	64.7
	Caucasian	T vs. C	1.09(0.88–1.34)	0.445	17.97	0.012	61.0
		TT + CT vs. CC	1.08(0.81–1.44)	0.616	18.02	0.012	61.2
		TT vs. CT + CC	1.07(0.86–1.34)	0.527	7.11	0.417	1.6
		TT vs. CC	1.20(0.83–1.73)	0.337	11.59	0.115	39.6
	African	T vs. C	0.98(0.72–1.32)	0.879	1.07	0.301	6.5
		TT + CT vs. CC	0.91(0.52–1.59)	0.735	1.95	0.162	48.8
		TT vs. CT + CC	1.57(0.80–3.09)	0.189	0.07	0.788	0
		TT vs. CC	1.30(0.65–2.63)	0.460	0.18	0.669	0
MTHFRA1298C	All studies	C vs. A	1.44(0.84–2.48)	0.191	35.80	<0.001	88.8
		CC + AC vs. AA	1.42(0.77–2.61)	0.263	26.32	<0.001	84.8
		CC vs. AC + AA	2.63(1.49–4.65)	0.001	7.55	0.109	47
		CC vs. AA	2.83(1.39–5.77)	0.004	10.27	0.036	61
	Caucasian	C vs. A	1.40(1.08–1.82)	0.011	1.83	0.4	0
		CC + AC vs. AA	1.39(0.97–1.98)	0.073	1.74	0.418	0
		CC vs. AC + AA	2.14(1.23–3.71)	0.007	1.68	0.433	0
		CC vs. AA	2.36(1.31–4.26)	0.004	1.58	0.454	0
	Asian	C vs. A	1.61(0.42–6.17)	0.484	23.67	<0.001	95.8
		CC + AC vs. AA	1.61(0.39–6.68)	0.513	20.23	<0.001	95.1
		CC vs. AC + AA	2.93(0.76–11.29)	0.118	2.16	0.142	53.7
		CC vs. AA	3.13(0.53–18.66)	0.210	3.34	0.068	70

Subgroup analysis by ethnicity revealed a substantial correlation between the MTHFR C677T polymorphism and MD in Asian populations (for T vs. C: OR = 1.46, 95% CI = 1.21–1.77, *P* < 0.001; for TT + CT vs. CC: OR = 1.52, 95% CI = 1.11–2.08, *P* = 0.009; for TT vs. CT + CC: OR = 1.75, 95% CI = 1.34–2.28, *P* < 0.001; for TT vs. CC: OR = 1.89, 95% CI = 1.40–2.57, *P* < 0.001), but not in Caucasian and African populations (Figure 4 and Table 4).

**FIGURE 5:**
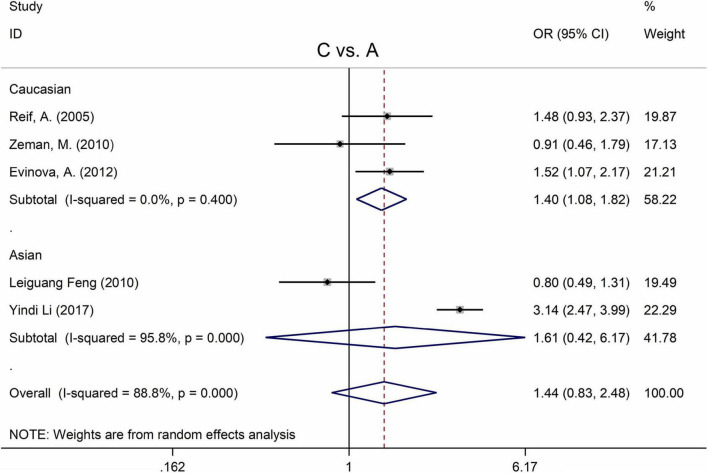
Forest plots for the associations between MTHFR A1298C polymorphisms and MD for the allele model with random effect model.

The MTHFR A1298C polymorphism was found to be highly associated with MD in the recessive model (for CC vs. AC + AA: OR = 2.63, 95% CI: 1.49–4.65, *P* = 0.001) and the homozygote model (for CC vs. AA: OR = 2.83, 95% Cl = 1.39–5.77, *P* = 0.004) (Table 4). Moreover, subgroup analysis demonstrated a positive correlation between the MTHFR A1298C polymorphism and MD in the Caucasian population (for C vs. A: OR = 1.40, 95% CI = 1.08–1.82, *P* = 0.011; for CC vs. AC + AA: OR = 2.14, 95% CI = 1.23–3.71, *P* = 0.007; for CC vs. AA: OR = 2.36, 95% CI = 1.31–4.26, *P* = 0.004) (Figure 5 and Table 4). Nonetheless, there was no statistical correlation between A1298C polymorphism and MD in Asian populations (Figure 5 and Table 4). Subgroup analysis shows that the correlation between MTHFR C677T polymorphism and MD exists in the Asian population (all genetic models) but not in Caucasian and African populations.

Four articles were not found in Hardy–Weinberg equilibrium (71, 84, 85, 95) (Tables 1, 2). Sensitivity analysis revealed that the overall correlation between MTHFR C677T polymorphism and MD remained unchanged after eliminating these data from the meta-analysis (Supplementary Figure 8). Sensitivity analyses for MTHFR A1298C polymorphism revealed that excluding Reif A. et al. (71) and Li et al. (95) resulted in a decreasing statistical correlation with MD; nonetheless, all statistical models revealed that MTHFR A1298C polymorphism was not significantly correlated with MD (Supplementary Figure 9).

#### Association between the methylenetetrahydrofolatereductase C677T/A1298C polymorphisms and bipolar disorder

**FIGURE 6:**
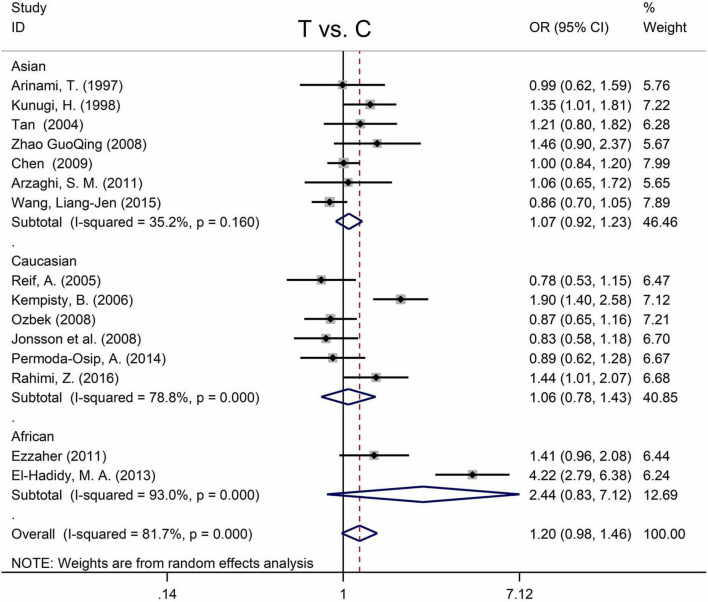
Forest plots for the associations between MTHFR C677T polymorphisms and BPD for the allele model with random effect model.

**FIGURE 7:**
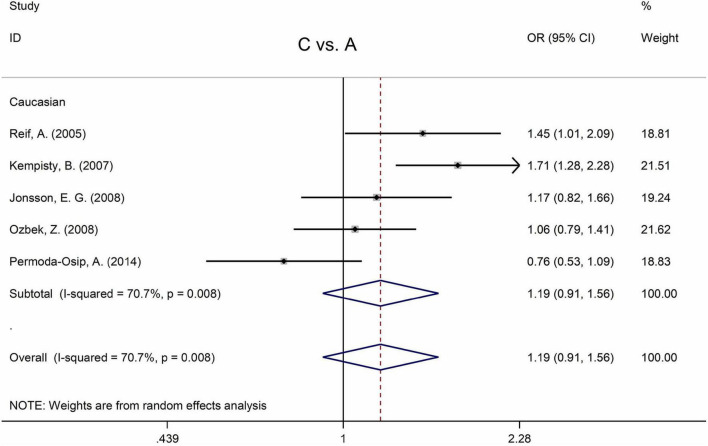
Forest plots for the associations between MTHFR A1298C polymorphisms and BPD for the allele model with random effect model.

Table 5 displays the main results and the heterogeneity test. There was a marginal correlation between the MTHFR C677T polymorphism and BPD in the recessive model (for TT vs. CT + CC: OR = 1.31, 95% CI: 1.03–1.67, *P* = 0.028) and the homozygote model (for TT vs. CC: OR = 1.40, 95% Cl = 1.00–1.94, *P* = 0.049) (Table 5). Moreover, subgroup analysis indicated no statistical correlation between the MTHFR C677T polymorphism and BPD in Asian, African, or Caucasian populations (Figure 6 and Table 5). Additionally, all models revealed that the MTHFR A1298C polymorphism was not statistically correlated with BPD (Figure 7 and Table 5).

**TABLE 5:** Odds ratios and heterogeneity results for the 4 genetic models of the MTHFR C677T and A1298C for BPD.

MTHFR		Comparison model	OR (95% CI)	*P*_*OR*_	Heterogeneity		
					*Q* within	*P*-value	*I*^2^ (%)
MTHFRC677T	All studies	T vs. C	1.20(0.98–1.46)	0.073	76.32	<0.001	81.7
		TT + CT vs. CC	1.21(0.93–1.57)	0.161	74.99	<0.001	81.3
		TT vs. CT + CC	1.31(1.03–1.67)	0.028	22.71	0.065	38.4
		TT vs. CC	1.40(1.00–1.94)	0.049	36.79	0.001	61.9
	Asian	T vs. C	1.07(0.92–1.24)	0.399	9.26	0.160	35.2
		TT + CT vs. CC	1.01(0.83–1.23)	0.926	8.6	0.197	30.2
		TT vs. CT + CC	1.23(0.99–1.54)	0.063	4.61	0.60	0
		TT vs. CC	1.17(0.91–1.49)	0.216	6.03	0.42	0.4
	Caucasian	T vs. C	1.06(0.78–1.43)	0.711	23.61	<0.001	78.8
		TT + CT vs. CC	1.03(0.73–1.46)	0.862	18.96	0.002	73.6
		TT vs. CT + CC	1.20(0.73–1.98)	0.468	11.67	0.04	57.2
		TT vs. CC	1.19(0.65–2.18)	0.566	15.96	0.007	68.7
	African	T vs. C	2.44(0.83–7.12)	0.104	14.29	<0.001	93.0
		TT + CT vs. CC	3.09(0.79–12.18)	0.106	14.15	<0.001	92.9
		TT vs. CT + CC	2.50(0.87–7.19)	0.09	2.59	0.107	61.4
		TT vs. CC	3.90(0.81–18.69)	0.089	5.29	0.021	81.1
MTHFRA1298C	All studies (Caucasian)	C vs. A	1.19(0.91–1.56)	0.208	13.67	0.008	70.7
		CC + AC vs. AA	1.19(0.91–1.56)	0.200	7.54	0.110	0.110
		CC vs. AC + AA	1.50(0.81–2.77)	0.200	13.66	0.008	70.7
		CC vs. AA	1.58(0.79–3.16)	0.200	15.85	0.003	74.8

Only one study was not in Hardy Weinberg equilibrium (71) (Table 2), and there was no statistical association between A1298C polymorphism and BPD after removing this study (Supplementary Figure 10).

#### Association between the methylenetetrahydrofolatereductase C677T/A1298C polymorphisms and psychiatric disorders

Significant publication biases were found when all diseases were considered (Supplementary Figure 5 and Supplementary Table 2). Therefore, analyses between MTHFR C677T and mental disorders were unsuitable here. However, the main results and the heterogeneity tests between MTHFR C677T and mental disorders were shown in Supplementary Table 1. Furthermore, the forest plots indicated that MTHFR C677T was strongly associated with psychiatric disorders, and sensitivity analysis did not affect the results (Supplementary Figures 1, 2).

Most studies were not in Hardy–Weinberg equilibrium when all diseases were considered. Moreover, analysis between MTHFR A1298C and psychiatric disorders was also unsuitable. Significant correlations were detected between the MTHFR A1298C polymorphism and psychiatric disorders (Supplementary Figure 3). However, sensitivity analysis revealed that excluding did change the conclusion (Supplementary Figure 4).

### Publication bias

**FIGURE 8:**
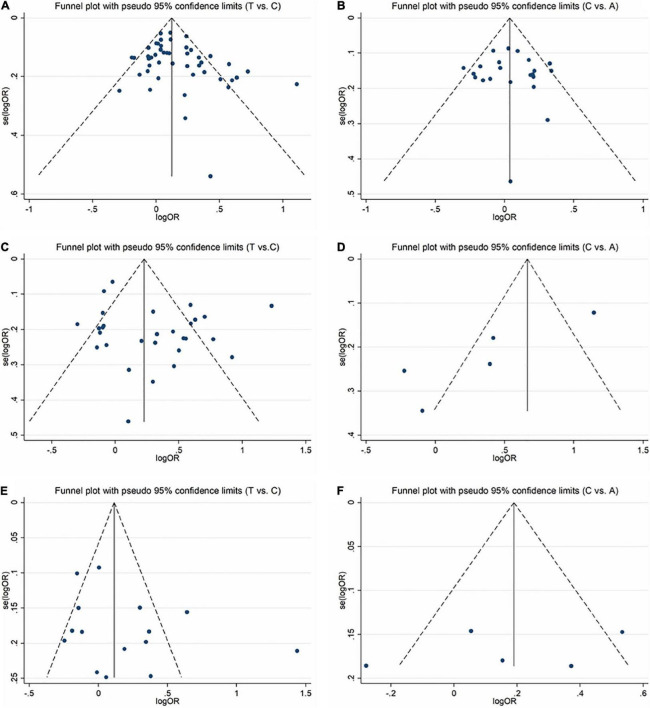
Funnel plots for assessing the publication bias risk in this meta-analysis. **(A)** Funnel plot for allele contrast (T vs. C) of C677T polymorphism in SZ. **(B)** Funnel plot for allele contrast (C vs. A) of A1298C polymorphism in SZ. **(C)** Funnel plot for allele contrast (T vs. C) of C677T polymorphism in MD. **(D)** Funnel plot for allele contrast (C vs. A) of A1298C polymorphism in MD. **(E)** Funnel plot for allele contrast (T vs. C) of C677T polymorphism in BPD. **(F)** Funnel plot for allele contrast (C vs. A) of A1298C polymorphism in BPD.

In order to evaluate publication bias, we used formal statistical methods (Egger’s regression test). Table 6 and Figure 8 presented the funnel plots for the meta-analysis. We observed that for SZ, no publication bias could be observed except in the dominant model (TT + CT vs. CC, *P*_Egger_ = 0.01). The Egger’s test results for MD were substantial in two genetic models of overall populations (allele model: C vs. A, *P*_Egger_ = 0.03; homozygote model: CC vs. AA, *P*_Egger_ = 0.02). And there was no publication bias for BPD. Publication bias may correlate to the editor’s decision for publication. However, it is common that only the positive results are published, and negative findings are unavailable. So, we could not exclude this kind of possibility.

**TABLE 6:** Publication bias risk in this meta-analysis.

Disease	MTHFR		*P*_Egger_	95% CL
Schizophrenia	C677T	T vs. C	0.05	0.02-2.10
		TT + CT vs. CC	0.01	0.27-2.08
		TT vs. CT + CC	0.24	−0.34-1.32
		TT vs. CC	0.06	−0.05-1.86
	A1298C	C vs. A	0.73	−1.60-2.25
		CC + AC vs. AA	0.66	−2.03-1.31
		CC vs. AC + AA	0.09	−0.17-2.39
		CC vs. AA	0.14	−0.35-2.32
Major depression	C677T	T vs. C	0.18	−0.64-3.33
		TT + CT vs. CC	0.35	−1.09-3.00
		TT vs. CT + CC	0.40	−0.80-1.97
		TT vs. CC	0.09	−0.21-2.52
	A1298C	C vs. A	0.03	−12.82–1.28
		CC + AC vs. AA	0.08	−13.00-1.10
		CC vs. AC + AA	0.05	−4.48-0.02
		CC vs. AA	0.02	−4.78–0.81
Bipolar disorder	C677T	T vs. C	0.19	−1.45-6.66
		TT + CT vs. CC	0.21	−1.62-6.66
		TT vs. CT + CC	0.31	−0.95-2.81
		TT vs. CC	0.18	−0.82-4.02
	A1298C	C vs. A	0.54	−30.72-19.79
		CC + AC vs. AA	0.54	−17.02-10.90
		CC vs. AC + AA	0.75	−24.98-31.11
		CC vs. AA	0.82	−26.75-31.41

## Discussion

A mental disorder is a neurological disease with complicated etiology, which may be closely related to genetic factors. A great number of research on the susceptibility to mental illnesses (including SZ, MD, and BPD) have been undertaken using MTHFR gene polymorphism. Some studies supported the susceptibility variation of MTHFR in mental diseases (27, 31, 70, 96, 105, 69), whereas other studies showed a negative correlation (28, 29, 33, 46, 102, 46). These variations might be due to the type of disease, ethnicity, or sample size. Our meta-analysis incorporates all previous research and provides more reliable evidence for the association between mental illness and MTHFR SNPs.

For Sz, our meta-analysis found a substantial association between MTHFR C677T polymorphism and higher incidence of SZ, which is consistent with research by Hu et al. (22) and Peerbooms et al. (23). In addition, we found that MTHFR A1298C polymorphism was not correlated with increased SZ risk, which is consistent with Peerbooms et al. (23). However, Hu et al. (22) discovered a marginal correlation between the MTHFR A1298C polymorphism and SZ. The inconsistency may be mainly owing to the limited sample size in the previous meta-analyses. For MTHFR C677T and A1298C, the sensitivity analysis has no substantial change to the results. As a result, the study’s findings are relatively consistent.

For MD, our meta-analysis’s results showed a significant correlation between MTHFR C677T polymorphism and increased risk of MD. The meta-analysis of Wu et al. (107) supports our view, whereas Gaysina et al. (108) and Peerbooms et al. (23) discovered no association between the C677T and MD. These discrepancies might be due to ethnicity, sample size, and other factors. Sensitivity analysis showed no change in the overall correlation between C677T polymorphism and MD. Also, we found a correlation between the A1298C polymorphism and MD (in recessive models and homozygous models). However, after excluding two studies not in Hardy–Weinberg equilibrium (38, 95), we discovered that A1298C polymorphism was not correlated with depression. We suspect that the reason for this is the insufficient number of studies included.

For BPD, meta-analysis reveals that MTHFR C677T polymorphism is weakly related to the occurrence of diseases (in recessive models and homozygous models). The meta-analysis of Hu et al. (22) found a marginal connection of C677T with an elevated risk of BPD (the recessive model), but some studies (21, 109, 110) found no associations. Different numbers of studies included may cause the inconsistency. Our meta-analysis included all current research, providing more reliable evidence for the association between MTHFR C677T polymorphism and BPD. As for A1298C, we only found studies in Caucasian people, and we did not find any association in these studies. The sensitivity analysis has no change to the results. Therefore, the results of this study are generally robust.

Many researchers have discovered that MTHFR is closely related to cognitive function, such as verbal fluency, visual-motor coordination, attention selectivity, and distribution (111–113). MTHFR polymorphism may also cause central nerve injury and microvascular injury, affect the synthesis of central neurotransmitters and the methylation of central neural system amines and phospholipids, and eventually lead to various mental diseases (114). All these impairments are not specific to one disease; therefore, we guess MTHFR may work on the common pathogenesis of these psychiatric disorders.

Some limitations of this meta-analysis should be considered when interpreting the findings. Firstly, we can only search for English and Chinese articles with some language limitations. Second, publication bias cannot be ignored in the current study since Egger test findings are substantial in several SZ and MD genetic models. It may correlate to the editor’s decision for publication and so on. However, it is common that only the positive results are published, and negative findings are unavailable. And we could not exclude this kind of possibility. Furthermore, the number of articles on A1298C polymorphism with MD are insufficient to provide conclusive evidence. More original research is required to validate our results. Despite some limitations, our current research also has some value. First of all, our meta-analysis includes a large sample size, which can reduce errors. Secondly, we fully considered and analyzed the impact of race on the disease.

## Conclusion

Our meta-analysis findings demonstrate that MTHFR C677T polymorphism increases the risk of schizophrenia and severe depression in the general population, and a marginal correlation of MTHFR C677T with a higher risk of bipolar disorder has also been reported for the recessive model. More original research and a bigger sample size are required to validate our results. Nevertheless, the findings of our meta-analysis imply that MTHFR may play a significant role in the common pathogenesis of mental illness and that its variation may be involved in controlling the expression of genes associated with it. It would help in the early diagnosis and treatment of related mental disorders. Moreover, studies on risk factor analysis could be performed on psychiatric disorders to better prevent these mental health problems.

## Data availability statement

The original contributions presented in this study are included in the article/Supplementary material, further inquiries can be directed to the corresponding author.

## Author contributions

Y-XZ: conceptualization, software, data curation, and writing – original draft preparation. DH: conceptualization, methodology, and funding acquisition. L-PY: data curation and validation. CG: visualization and investigation. C-CC: software and validation. Z-YG: writing – reviewing and editing. H-MS: project administration and supervision. All authors contributed to the article and approved the submitted version.
