# Supplementary material for: Association between variants of MTHFR genes and psychiatric disorders: A meta-analysis
Source: Front Psychiatry. 2022 Aug 18;13:976428. doi: 10.3389/fpsyt.2022.976428 (PMC9433753; doi:10.3389/fpsyt.2022.976428)
Supplement: Supplementary file 1 [file Data_Sheet_1.docx]

Supplementary materials for

**Association between variants of MTHFR genes and** **psychiatric disorders: a meta-analysis**

Yu-Xin Zhang ^a,b,†^ , Lu-Ping Yang ^c,†^, Cong Gai ^a^, Cui-Cui Cheng ^a^, Jin-Kun Zhang ^a^, Hong-Mei Sun ^a^, Die Hu ^a,^^*^

^a^ Department of Anatomy, School of Preclinical Medicine, Beijing University of Chinese Medicine, Beijing, China

^b^ Beijing Research Institute of Chinese Medicine, Beijing University of Chinese Medicine, Beijing, China

^c^ School of Chinese Materia Medica, Beijing University of Chinese Medicine, Beijing, China

^†^ These authors have contributed equally to this work

^*^ Corresponding author:

E-mail addresses: hudie0610@bucm.edu.cn (D Hu);.

Current Address: Department of Anatomy, School of Preclinical Medicine, Beijing University of Chinese Medicine, Sunshine south street, Beijing 102488, China.

**This file includes:**

Table S1 – S2;

Supplementary Fig S1 - S10.

**Table S1**

Odds ratios and heterogeneity results for the 4 genetic models of the MTHFR C677T/A1298C for psychiatric disorders.

|  |  | Comparison model | OR (95 % CI) | P_OR_ | Heterogeneity | | |
| --- | --- | --- | --- | --- | --- | --- | --- |
|  |  |  |  |  | Q within | P value | I^2^ (%) |
| MTHFR  C677T | All studies | T vs. C | 1.23(1.16-1.31) | <0.001 | 361.04 | <0.001 | 75.9 |
|  |  | TT + CT vs. CC | 1.26(1.16-1.37) | <0.001 | 365.30 | <0.001 | 74.8 |
|  |  | TT vs. CT + CC | 1.35(1.23-1.47) | <0.001 | 187.99 | <0.001 | 51.1 |
|  |  | TT vs. CC | 1.46(1.31-1.63) | <0.001 | 242.81 | <0.001 | 62.1 |
|  | Asian | T vs. C | 1.30(1.20-1.41) | <0.001 | 224.39 | <0.001 | 76.8 |
|  |  | TT + CT vs. CC | 1.35(1.20-1.53) | <0.001 | 227.17 | <0.001 | 77.1 |
|  |  | TT vs. CT + CC | 1.46(1.30-1.64) | <0.001 | 116.34 | <0.001 | 55.3 |
|  |  | TT vs. CC | 1.63(1.40-1.88) | <0.001 | 147.45 | <0.001 | 64.7 |
|  | Caucasian | T vs. C | 1.08(1.00-1.17) | 0.039 | 77.71 | <0.001 | 57.5 |
|  |  | TT + CT vs. CC | 1.09(0.99-1.19) | 0.069 | 66.42 | <0.001 | 50.3 |
|  |  | TT vs. CT + CC | 1.13(1.00-1.27) | 0.060 | 46.76 | 0.057 | 29.4 |
|  |  | TT vs. CC | 1.16(1.00-1.35) | 0.045 | 59.91 | 0.003 | 44.9 |
|  | African | T vs. C | 1.82(1.04-3.18) | 0.036 | 43.16 | <0.001 | 88.4 |
|  |  | TT + CT vs. CC | 1.86(0.90-3.84) | 0.095 | 47.15 | <0.001 | 89.4 |
|  |  | TT vs. CT + CC | 2.38(1.47-3.85) | <0.001 | 5.99 | 0.307 | 16.5 |
|  |  | TT vs. CC | 3.10(1.42-6.74) | 0.004 | 12.85 | 0.025 | 61.1 |
| MTHFR  A1298C | All studies | C vs. A | 1.12(1.00-1.26) | 0.041 | 132.75 | <0.001 | 76.6 |
|  |  | CC + AC vs. AA | 1.13(1.01-1.26) | 0.039 | 81.13 | <0.001 | 61.8 |
|  |  | CC vs. AC + AA | 1.28(1.03-1.60) | 0.029 | 94.40 | <0.001 | 67.2 |
|  |  | CC vs. AA | 1.34(1.05-1.71) | 0.021 | 107.55 | <0.001 | 71.2 |
|  | Caucasian | C vs. A | 1.12(1.01-1.24) | 0.034 | 35.42 | <0.001 | 52.0 |
|  |  | CC + AC vs. AA | 1.12(1.00-1.25) | 0.045 | 22.91 | 0.152 | 25.8 |
|  |  | CC vs. AC + AA | 1.29(1.02-1.62) | 0.033 | 36.28 | 0.004 | 53.1 |
|  |  | CC vs. AA | 1.33(1.04-1.72) | 0.026 | 40.08 | 0.001 | 57.6 |
|  | Asian | C vs. A | 1.13(0.90-1.42) | 0.311 | 96.87 | <0.001 | 86.6 |
|  |  | CC + AC vs. AA | 1.14(0.91-1.41) | 0.255 | 58.08 | <0.001 | 77.6 |
|  |  | CC vs. AC + AA | 1.25(0.79-1.98) | 0.344 | 57.92 | <0.001 | 77.6 |
|  |  | CC vs. AA | 1.32(1.05-1.71) | 0.284 | 67.01 | <0.001 | 80.6 |

**Table S2**

Publication bias risk between MTHFR and psychiatric disorders in this meta-analysis.

| MTHFR | | P_egger_ | 95%CL |
| --- | --- | --- | --- |
| C677T | T vs. C | 0.003 | 0.49－2.29 |
|  | TT + CT vs. CC | 0.005 | 0.40－2.15 |
|  | TT vs. CT + CC | 0.30 | 0.64－1.27 |
|  | TT vs. CC | 0.002 | 0.39－1.77 |
| A1298C | C vs. A | 0.95 | -2.59－2.42 |
|  | CC + AC vs. AA | 0.68 | -2.36－1.56 |
|  | CC vs. AC + AA | 0.39 | -0.92－2.28 |
|  | CC vs. AA | 0.55 | -1.22－2.26 |

Fig. S1. Forest plots for the associations between MTHFR C677T polymorphisms and psychiatric disorders for the allele model with random effect model.

Fig. S2. After excluded the study not in Hardy–Weinberg equilibrium , forest plots for the associations between MTHFR C677T polymorphisms and psychiatric disorders for the allele model with random effect model.

Fig. S3. Forest plots for the associations between MTHFR A1298C polymorphisms and psychiatric disorders for the allele model with random effect model.

Fig. S4. After excluded the study not in Hardy–Weinberg equilibrium , forest plots for the associations between MTHFR A1298C polymorphisms and psychiatric disorders for the allele model with random effect model.

b

a

Fig. S5. Funnel plots for assessing the publication bias risk in this meta-analysis.

**a** Funnel plot for allele contrast (T vs. C) of C677T polymorphism in psychiatric disorders;

**b** Funnel plot for allele contrast (C vs. A) of A1298C polymorphism in psychiatric disorders.

Fig. S6. After excluded the study not in Hardy–Weinberg equilibrium , forest plots for the associations between MTHFR C677T polymorphisms and SZ for the allele model with random effect model

Fig. S7. After excluded the study not in Hardy–Weinberg equilibrium , forest plots for the associations between MTHFR A1298C polymorphisms and SZ for the allele model with random effect model

Fig. S8. After excluded the study not in Hardy–Weinberg equilibrium, forest plots for the associations between MTHFR C677T polymorphisms and MD for the allele model with random effect model

Fig. S9. After excluded the study not in Hardy–Weinberg equilibrium , forest plots for the associations between MTHFR A1298C polymorphisms and MD for the allele model with random effect model

Fig. S10. After excluded the study not in Hardy–Weinberg equilibrium , forest plots for the associations between MTHFR A1298C polymorphisms and BPD for the allele model with random effect model
